# Supplementary material for: Exciton-to-trion conversion as a control mechanism for valley polarization in room-temperature monolayer WS2
Source: Sci Rep. 2020 Oct 15;10:17389. doi: 10.1038/s41598-020-74376-3 (PMC7566459; doi:10.1038/s41598-020-74376-3)
Supplement: Supplementary file 1 — Supplementary Information. [file 41598_2020_74376_MOESM1_ESM.pdf]

Supplementary Information

Exciton-to-trion conversion as a control mechanism for valley polarization in room-temperature monolayer WS_2

Joris J. Carmiggelt,[†,‡] Michael Borst,[†,‡] and Toeno van der Sar[*,†]

†*Department of Quantum Nanoscience, Kavli Institute of Nanoscience, Delft University of Technology, Lorentzweg 1, 2628 CJ Delft, The Netherlands*

‡*These authors contributed equally to this work*

E-mail: t.vandersar@tudelft.nl

Section S1: Literature values of chemical potential and hardness

Table S1: Chemical potential and chemical hardness of anisole and monolayer WS_2 calculated using density functional theory.

	WS_2 monolayer[1]	Anisole[2]
Chemical potential μ (eV)	-4.79	-3.17
Chemical hardness η (eV)	2.64	4.88

Section S2: Reproducable quenching-induced valley polarization

We repeat the anisole treatment as specified in the main text on five more WS_2 monolayer flakes, presented in Figure S2, all of which show a strong dimming and emergent room-temperature valley polarization after doping. Of these, three have a Si/SiO_2 substrate and

two an yttrium iron garnet (YIG) substrate. Variations in photoluminescence and valley polarization between the flakes are attributed to differences in defect density and unintentional doping prior to the treatment. Furthermore, we confirm the absence of valley polarization before the anisole treatment by presenting spectra of untreated monolayers for different excitation and detection helicities in Figure S2.

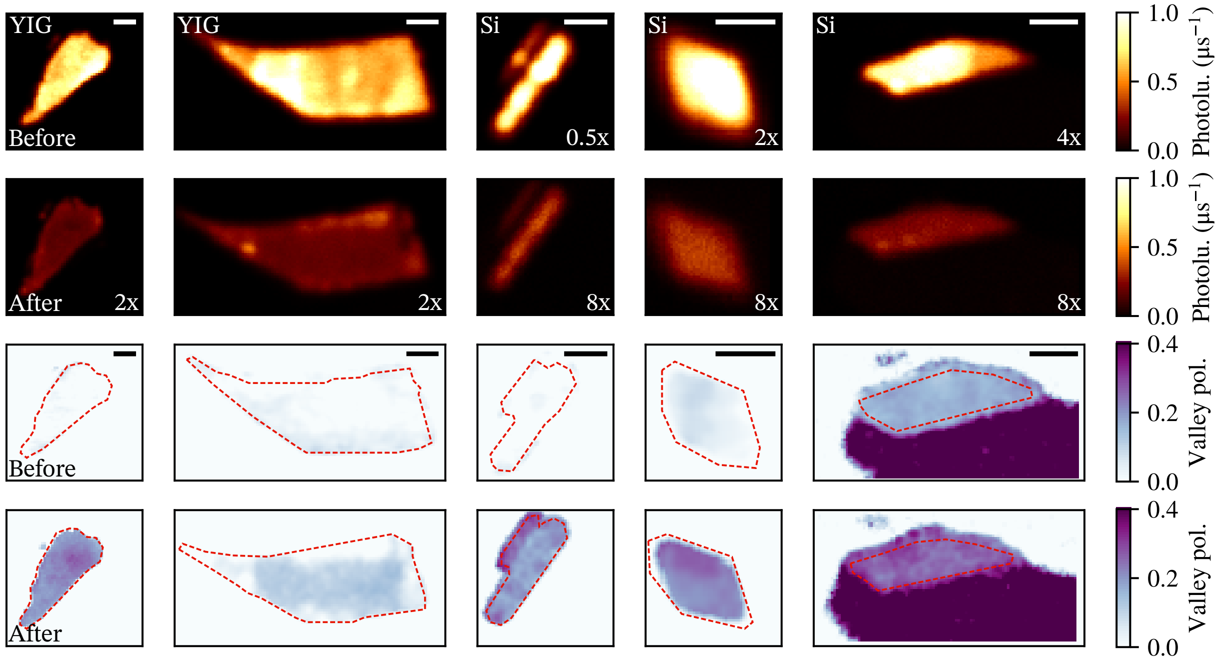

Figure S1: Additional photoluminescence and valley polarization maps of WS_2 monolayer flakes before and after anisole treatment. The first two monolayers were stamped on yttrium iron garnet (YIG) and the remaining three on Si/SiO_2. All maps were made using a 594 nm excitation laser at a power of 4 μW before the doping and 40 μW after doping. Scale bar: 2 μm.

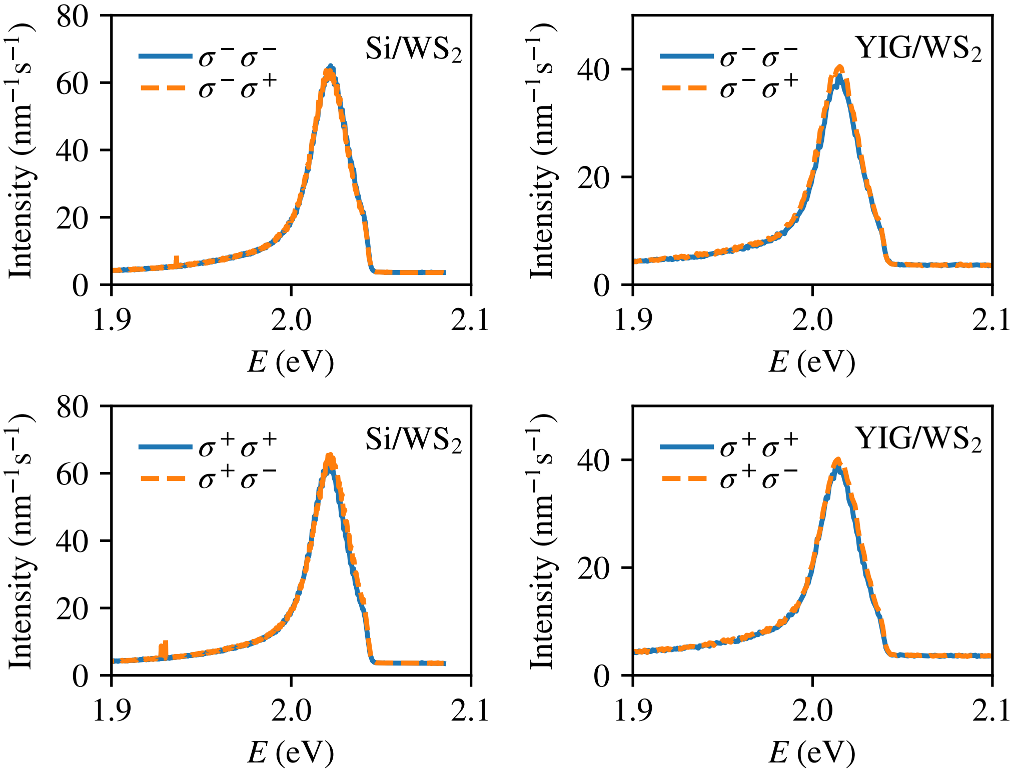

Figure S2: σ^+ and σ^- emission spectra under σ^+ and σ^- near-resonance excitation (E = 2.087 eV, λ = 594 nm) of untreated WS_2 monolayers on Si/SiO_2 and YIG substrates. The legend entry $\sigma^+\sigma^-$ corresponds to exciting with σ^+ and detecting with σ^-. In each panel σ^+ and σ^- emission spectra are overlapping, which confirms the absence of valley polarization in these flakes. At about 2.04 eV the spectra are cut by the longpass filters that block the excitation laser.

Section S3: Emergence of exciton emission at wrinkles

Here, we plot additional σ^+ emission spectra along spatial traces over wrinkles in the WS_2 flake presented in Figure 3 of the main text. As demonstrated in Figure S3, each trace is characterized by the emergence of a strong exciton resonance at the center of the wrinkle, highlighted by the asymmetry of the spectra. The central wavelength of both trions and excitons varies slightly over the different wrinkles, which we attribute to local variations in strain[3,4] and doping.[5] From this, we extract spatially varying energy splittings between the excitons and trions within the range of 22 meV − 32 meV, in agreement with reported

literature values.[6] Additional σ^- emission spectra were taken along the same spatial traces to determine the valley polarization and brightness of the exciton and trion resonances near wrinkles, which were plotted in Figure 3e of the main text.

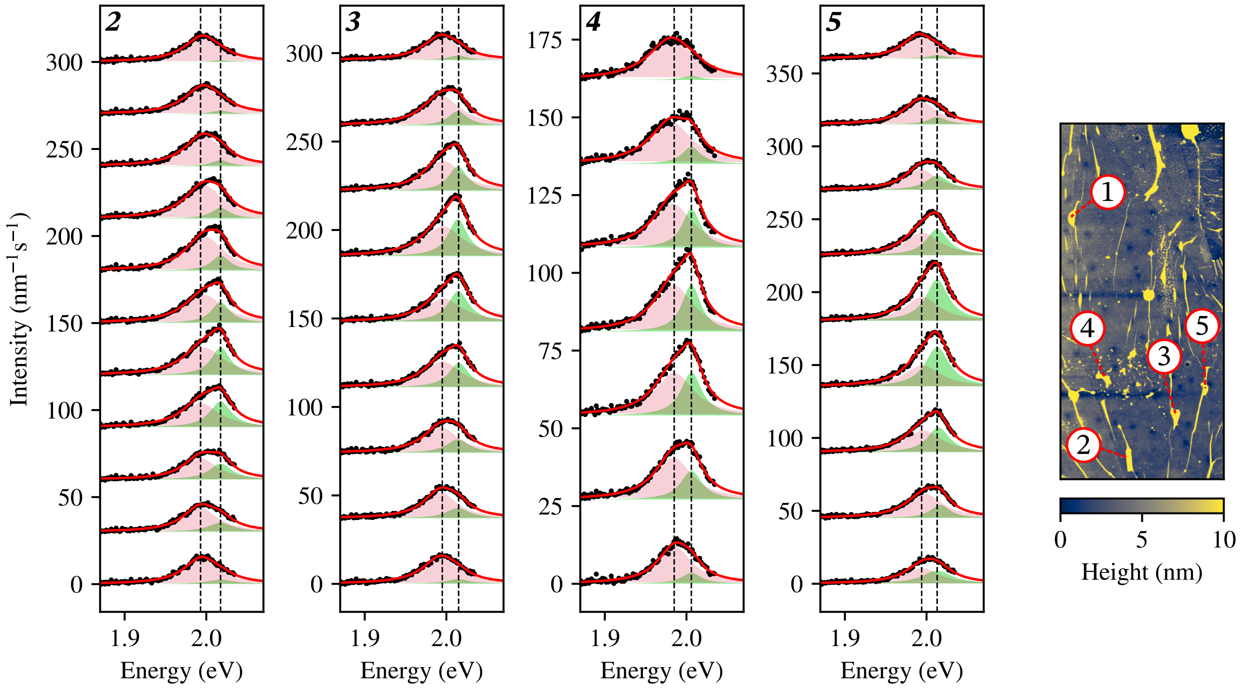

Figure S3: Recurrent emergence of exciton emission at wrinkles. σ^+ emission spectra are plotted along spatial traces over multiple wrinkles on the sample of Figure 3 of the main text. Labels above the spectra correspond to wrinkles in the atomic force microscope map in the right panel. Emission spectra at label 1 are depicted in Figure 3c of the main text. All spectra were taken at near-resonance excitation ($E = 2.087$ eV, $\lambda = 594$ nm, 40 µW).

Section S4: Modelling doping-controlled valley dynamics

S4.1 Model overview

To calculate the expected valley polarization as a function of doping level we extend the rate equation model of Lien et al.[7] by incorporating the valleys (Figure S4). The model assumes that excitons are excited in the K valley at a rate Γ_{K}, scatter to the K' valley at a rate $\Gamma_{\mathrm{iv,X}}$, decay radiatively at a rate $\Gamma_{\mathrm{r,X}}$, or decay to trions via $\Gamma_{\mathrm{T}\leftarrow\mathrm{X}}$ while preserving their valley. The trions then scatter between the valleys at a rate $\Gamma_{\mathrm{iv,T}}$, decay radiatively via $\Gamma_{\mathrm{r,T}}$,

non-radiatively via Γ_{nr}, or back to excitons via $\Gamma_{\mathrm{X}\leftarrow\mathrm{T}}$ while preserving their valley.

A law of mass-action states that $\Gamma_{\mathrm{T}\leftarrow\mathrm{X}}$ is linearly proportional to the electron density n_e, while $\Gamma_{\mathrm{X}\leftarrow\mathrm{T}}$ is fixed.[7–9] As described in the main text, we expect that $\Gamma_{\mathrm{T}\leftarrow\mathrm{X}}$ is spatially fluctuating due to local variations in doping level. Since the samples are strongly doped, we assume that $\Gamma_{\mathrm{T}\leftarrow\mathrm{X}}$ is much larger than any other non-radiative decay rate of the excitons, which we therefore neglect.

We define the probability for the system to be in the ground state as G, the probability to have formed an exciton in the $\mathrm{K}^{(\text{'})}$ valley as $X_{\mathrm{K}^{(\text{'})}}$, and the probability to have formed a trion in the $\mathrm{K}^{(\text{'})}$ valley as $T_{\mathrm{K}^{(\text{'})}}$. The time evolution of the system is then governed by the following master equations:

$$\frac{dG}{dt} = -\Gamma_{\mathrm{K}}G + \Gamma_{\mathrm{r,X}}(X_{\mathrm{K}} + X_{\mathrm{K'}}) + (\Gamma_{\mathrm{r,T}} + \Gamma_{\mathrm{nr}})(T_{\mathrm{K}} + T_{\mathrm{K'}}) = 0, \tag{1}$$

$$\frac{dX_{\mathrm{K}}}{dt} = \Gamma_{\mathrm{K}}G - (\Gamma_{\mathrm{iv,X}} + \Gamma_{\mathrm{T}\leftarrow\mathrm{X}} + \Gamma_{\mathrm{r,X}})X_{\mathrm{K}} + \Gamma_{\mathrm{iv,X}}X_{\mathrm{K'}} + \Gamma_{\mathrm{X}\leftarrow\mathrm{T}}T_{\mathrm{K}} = 0, \tag{2}$$

$$\frac{dX_{\mathrm{K'}}}{dt} = -(\Gamma_{\mathrm{iv,X}} + \Gamma_{\mathrm{T}\leftarrow\mathrm{X}} + \Gamma_{\mathrm{r,X}})X_{\mathrm{K'}} + \Gamma_{\mathrm{iv,X}}X_{\mathrm{K}} + \Gamma_{\mathrm{X}\leftarrow\mathrm{T}}T_{\mathrm{K'}} = 0, \tag{3}$$

$$\frac{dT_{\mathrm{K}}}{dt} = -(\Gamma_{\mathrm{iv,T}} + \Gamma_{\mathrm{X}\leftarrow\mathrm{T}} + \Gamma_{\mathrm{nr}} + \Gamma_{\mathrm{r,T}})T_{\mathrm{K}} + \Gamma_{\mathrm{iv,T}}T_{\mathrm{K'}} + \Gamma_{\mathrm{T}\leftarrow\mathrm{X}}X_{\mathrm{K}} = 0, \tag{4}$$

$$\frac{dT_{\mathrm{K'}}}{dt} = -(\Gamma_{\mathrm{iv,T}} + \Gamma_{\mathrm{X}\leftarrow\mathrm{T}} + \Gamma_{\mathrm{nr}} + \Gamma_{\mathrm{r,T}})T_{\mathrm{K'}} + \Gamma_{\mathrm{iv,T}}T_{\mathrm{K}} + \Gamma_{\mathrm{T}\leftarrow\mathrm{X}}X_{\mathrm{K'}} = 0, \tag{5}$$

$$G + X_{\mathrm{K}} + X_{\mathrm{K'}} + T_{\mathrm{K}} + T_{\mathrm{K'}} = 1. \tag{6}$$

Here, we set the time derivatives to zero to consider a steady state and finally normalize the probabilities in the last line.

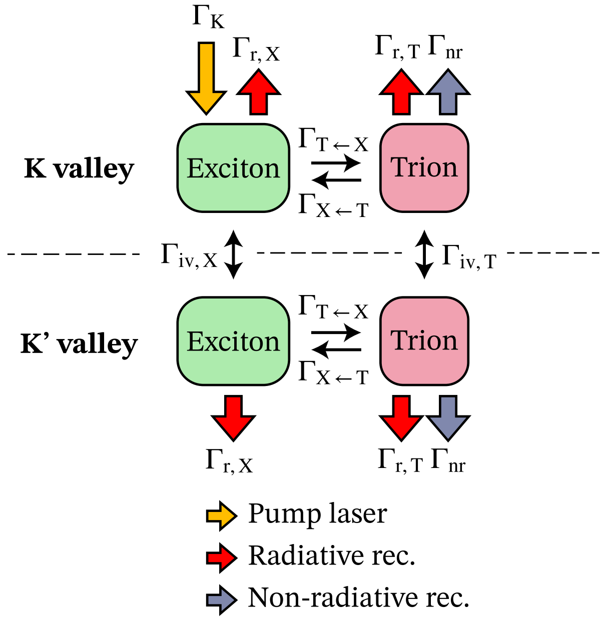

Figure S4: Schematic overview of the rate equation model. The closed system can either be in the ground state, or be an exciton or trion in the K or K' valley. By considering its steady state solutions, we model the valley polarization of excitons and trions under continuous-wave illumination.

S4.2 Calculation of the trion and exciton valley polarization

The valley polarization of the exciton and trion are respectively denoted by ρ_{X} and ρ_{T}, and are defined as the normalized asymmetry in valley occupation,

$$\begin{aligned} \rho_{\mathrm{X}} &= \frac{X_{\mathrm{K}} - X_{\mathrm{K'}}}{X_{\mathrm{K}} + X_{\mathrm{K'}}}, \\ \rho_{\mathrm{T}} &= \frac{T_{\mathrm{K}} - T_{\mathrm{K'}}}{T_{\mathrm{K}} + T_{\mathrm{K'}}}. \end{aligned} \tag{7}$$

By combining equations 4 and 5 we derive a relation between ρ_{X} and ρ_{T}, demonstrating that the valley polarization of excitons is always larger than that of trions[10]

$$\rho_{\mathrm{T}} = \frac{\Gamma_{\mathrm{X}\leftarrow\mathrm{T}} + \Gamma_{\mathrm{nr}} + \Gamma_{\mathrm{r,T}}}{2\Gamma_{\mathrm{iv,T}} + \Gamma_{\mathrm{X}\leftarrow\mathrm{T}} + \Gamma_{\mathrm{nr}} + \Gamma_{\mathrm{r,T}}}\rho_{\mathrm{X}}. \tag{8}$$

We can express ρ_X in terms of the rates by combining equations 1-5

$$\rho_X = \frac{\frac{\Gamma_{r,X}}{2\Gamma_{iv,X}} + \frac{\Gamma_{r,T}+\Gamma_{nr}}{2(\Gamma_{X\leftarrow T}+\Gamma_{nr}+\Gamma_{r,T})}\frac{\Gamma_{T\leftarrow X}}{\Gamma_{iv,X}}}{1+\frac{\Gamma_{r,X}}{2\Gamma_{iv,X}} + \frac{2\Gamma_{iv,T}+\Gamma_{nr}+\Gamma_{r,T}}{2(2\Gamma_{iv,T}+\Gamma_{X\leftarrow T}+\Gamma_{nr}+\Gamma_{r,T})}\frac{\Gamma_{T\leftarrow X}}{\Gamma_{iv,X}}} \approx \frac{\frac{\Gamma_{nr}}{2(\Gamma_{X\leftarrow T}+\Gamma_{nr})}\frac{\Gamma_{T\leftarrow X}}{\Gamma_{iv,X}}}{1+\frac{2\Gamma_{iv,T}+\Gamma_{nr}}{2(2\Gamma_{iv,T}+\Gamma_{X\leftarrow T}+\Gamma_{nr})}\frac{\Gamma_{T\leftarrow X}}{\Gamma_{iv,X}}}. \tag{9}$$

Since the radiative decay rates are known to be low at room temperature,[7,11,12] we assumed in the last step that $\Gamma_{iv,X} >> \Gamma_{r,X}$ and $\Gamma_{nr} >> \Gamma_{r,T}$. We thus conclude that the valley polarization of the excitons is parametrized by $\frac{\Gamma_{T\leftarrow X}}{\Gamma_{iv,X}}$, and that it reaches a maximum ρ_X^{max} when $\Gamma_{T\leftarrow X} >> \Gamma_{iv,X}$

$$\rho_X^{max} = \frac{\Gamma_{nr}(2\Gamma_{iv,T}+\Gamma_{X\leftarrow T}+\Gamma_{nr})}{(\Gamma_{nr}+2\Gamma_{iv,T})(\Gamma_{nr}+\Gamma_{X\leftarrow T})}. \tag{10}$$

S4.3 Analysis of the valley polarization versus photoluminescence

Using equations 4, 5 and 9 we can calculate the valley polarization ρ as it detected by the avalanche photodiode (APD) in the experiment, by summing the exciton and trion emission

$$\begin{aligned}\rho &= \frac{\Gamma_{r,T}(T_K - T_{K'}) + \Gamma_{r,X}(X_K - X_{K'})}{\Gamma_{r,T}(T_K + T_{K'}) + \Gamma_{r,X}(X_K + X_{K'})}\\ &= \frac{\frac{\Gamma_{r,T}}{\Gamma_{r,X}(2\Gamma_{iv,T}+\Gamma_{X\leftarrow T}+\Gamma_{nr})}\Gamma_{T\leftarrow X}+1}{\frac{\Gamma_{r,T}}{\Gamma_{r,X}(\Gamma_{X\leftarrow T}+\Gamma_{nr})}\Gamma_{T\leftarrow X}+1}\frac{\frac{\Gamma_{nr}}{2\Gamma_{iv,X}(\Gamma_{X\leftarrow T}+\Gamma_{nr})}\Gamma_{T\leftarrow X}}{\frac{2\Gamma_{iv,T}+\Gamma_{nr}}{2\Gamma_{iv,X}(2\Gamma_{iv,T}+\Gamma_{X\leftarrow T}+\Gamma_{nr})}\Gamma_{T\leftarrow X}+1}.\end{aligned} \tag{11}$$

To find a relation between the valley polarization and total photoluminescence $I = I_T + I_X$, we express I in terms of the rates by combining equations 1 and 4-6, and find that it is inversely related to $\Gamma_{T\leftarrow X}$

$$I = \alpha N\big(\Gamma_{r,T}(T_K + T_{K'}) + \Gamma_{r,X}(X_K + X_{K'})\big) \approx \alpha N\Gamma_K(\frac{\Gamma_{r,T}}{\Gamma_{nr}} + \frac{\Gamma_{r,X}(\frac{\Gamma_{X\leftarrow T}}{\Gamma_{nr}}+1)}{\Gamma_{T\leftarrow X}}). \tag{12}$$

Here $0 < \alpha < 1$ represents the finite detection efficiency of the confocal microscope and N is the number of electrons in the system. To simplify this expression, we made use of the previous assumptions, and assumed that the system is weakly excited and strongly doped, such that Γ_K is small and $\Gamma_{T\leftarrow X} >> \Gamma_{r,X}$. By inverting this equation and substituting it into

equation 11, we find ρ as a function of I for a sample with a varying doping level

$$\rho = \frac{a_0 I + 1}{b_0 I^2 + c_0 I}. \tag{13}$$

Here the constants are given by

$$\begin{aligned}
a_0 &= -\frac{\Gamma_{\mathrm{nr}}}{2\alpha N \Gamma_{\mathrm{K}} \Gamma_{\mathrm{iv,T}} \Gamma_{\mathrm{r,T}}} (2\Gamma_{\mathrm{iv,T}} + \Gamma_{\mathrm{X \leftarrow T}} + \Gamma_{\mathrm{nr}}) \\
b_0 &= -\frac{\Gamma_{\mathrm{nr}} \Gamma_{\mathrm{iv,X}}}{\alpha^2 N^2 \Gamma_{\mathrm{K}}^2 \Gamma_{\mathrm{r,X}} \Gamma_{\mathrm{r,T}} \Gamma_{\mathrm{iv,T}}} (2\Gamma_{\mathrm{iv,T}} + \Gamma_{\mathrm{X \leftarrow T}} + \Gamma_{\mathrm{nr}}) \\
c_0 &= -\frac{1}{2\alpha N \Gamma_{\mathrm{K}} \Gamma_{\mathrm{iv,T}} \Gamma_{\mathrm{r,T}}} (\Gamma_{\mathrm{X \leftarrow T}} + \Gamma_{\mathrm{nr}})(2\Gamma_{\mathrm{iv,T}} + \Gamma_{\mathrm{nr}}) - \frac{2\Gamma_{\mathrm{iv,X}} \Gamma_{\mathrm{r,T}}}{\Gamma_{\mathrm{r,X}} \Gamma_{\mathrm{nr}}} a_0
\end{aligned} \tag{14}$$

In agreement to our data, the valley polarization should thus be inversely related to the photoluminescence at high I

$$\rho \approx \frac{a_0}{b_0 I + c_0}. \tag{15}$$

This inverse relation is reinforced by the spatial averaging of our diffraction-limited optical spot (see section S5). At low I, a local maximum in the valley polarization is experimentally observed, only if

$$\frac{\Gamma_{\mathrm{nr}}}{2\Gamma_{\mathrm{r,T}}} \left(\frac{\Gamma_{\mathrm{r,X}}}{\Gamma_{\mathrm{iv,X}}} - \frac{\Gamma_{\mathrm{r,T}}}{\Gamma_{\mathrm{iv,T}}} \right) + \frac{\Gamma_{\mathrm{r,X}} \Gamma_{\mathrm{iv,T}}}{\Gamma_{\mathrm{iv,X}} \Gamma_{\mathrm{r,T}}} - \frac{\Gamma_{\mathrm{X \leftarrow T}}}{2\Gamma_{\mathrm{iv,T}}} > 1. \tag{16}$$

This is only true, if $\frac{\Gamma_{\mathrm{r,X}}}{\Gamma_{\mathrm{iv,X}}} > \frac{\Gamma_{\mathrm{r,T}}}{\Gamma_{\mathrm{iv,T}}}$, provided that $\Gamma_{\mathrm{iv,T}} >> \Gamma_{\mathrm{X \leftarrow T}}$. We thus conclude that relatively bright excitons with a significantly higher polarization than trions are required for the observation of a local maximum in the total valley polarization versus photoluminescence.

S4.4 Comparison between model and experiment

To better assess the correspondence between the model and the experimental data in Figure 4c of the main text, we make a basic estimate of the valley polarization using MoS_2 literature values for the radiative and non-radiative rates[7] ($1/\Gamma_{\mathrm{r,X}} = 8$ ns, $1/\Gamma_{\mathrm{r,T}} = 110$ ns and $1/\Gamma_{\mathrm{nr}} =$ 50 ps). For a rough approximation, we assume that $\Gamma_{\mathrm{X \leftarrow T}}$ is much smaller than the other rates. As a result, the exciton valley polarization approaches unity at strong doping via

equation 10, while the trion polarization is parametrized by $\Gamma_{\mathrm{iv,T}}$ via equation 8. Following the experimental results in Figure 4c, we tune the trion polarization at maximal doping to about 40%, which gives $1/\Gamma_{\mathrm{iv,T}} = 60$ ps. At this stage only $\Gamma_{\mathrm{iv,X}}$ remains as a free parameter and it determines whether the valley polarization has a local maximum as a function of photoluminescence via equation 16.

In Figure 4b of the main text we demonstrate that for $1/\Gamma_{\mathrm{iv,X}} = 10$ ps a small local maximum in valley polarization can be observed. In contrast, at an enhanced intervalley scattering of $1/\Gamma_{\mathrm{iv,X}} = 2$ ps the local maximum disappears (Figure S5, left panel), because $\frac{\Gamma_{\mathrm{r,X}}}{\Gamma_{\mathrm{iv,X}}} < \frac{\Gamma_{\mathrm{r,T}}}{\Gamma_{\mathrm{iv,T}}}$.

To compare the model and experiment, we plot the valley polarization versus photoluminescence using equation 13 for different values of $\Gamma_{\mathrm{iv,X}}$ (Figure S5, right panel). We note that we obtain similar plots when increasing $\Gamma_{\mathrm{X \leftarrow T}}$, which merely lowers the exciton valley polarization below unity at strong doping and decreases the difference between the trion and exciton polarization, making the presence of a local maximum less likely.

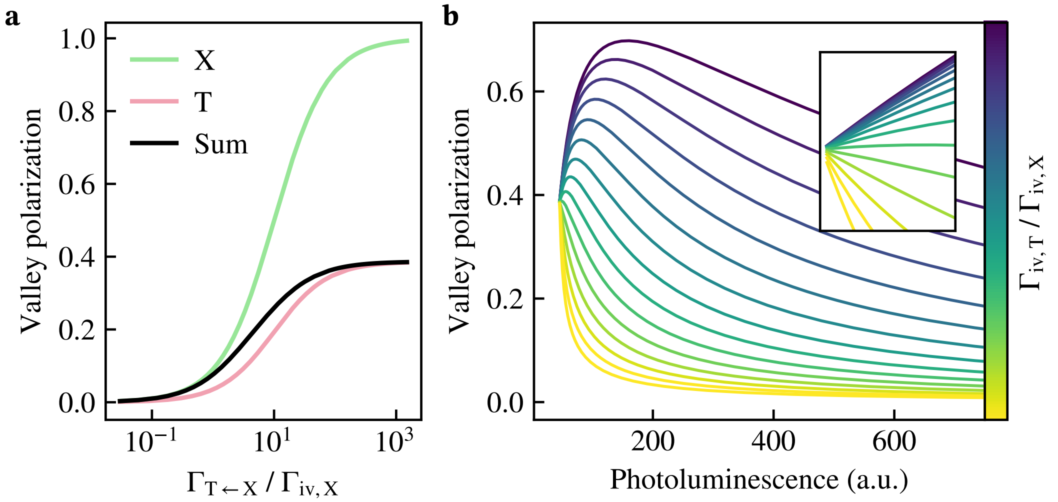

Figure S5: Modelling valley polarization as a function of trion-to-exciton conversion and total luminescence. (a) Modelled valley polarization versus $\Gamma_{\mathrm{T \leftarrow X}}/\Gamma_{\mathrm{iv,X}}$ using the same rates as in Figure 4b of the main text, demonstrating that a different choice of $\Gamma_{\mathrm{iv,X}}$ can yield absence of a local maximum. (b) Simulated valley polarization versus photoluminescence using the same rates as in (a), but with logarithmically spaced values for $1/\Gamma_{\mathrm{iv,X}}$ ranging between 1 and 100 ps. The inset highlights the presence (absence) of a local maximum at low brightness for low (high) $\Gamma_{\mathrm{iv,X}}$.

Section S5. Calculation of the spatially-averaged valley polarization of adjacent regions with different doping levels

In this section we calculate the valley polarization versus photoluminescence when averaging the emission of two regions with different doping levels. In particular, we consider a strongly-doped region with weak and highly polarized trion emission and a weakly-doped region with strong and weakly polarized exciton emission, similar to the emission detected on wrinkles in Figure 3 of the main text. The emission from both regions is collected by our diffraction-limited optical spot, leading to a spatially-averaged valley polarization of

$$\rho = \frac{\Gamma_{\mathrm{r,T}}(T_{\mathrm{K}} - T_{\mathrm{K'}}) + \Gamma_{\mathrm{r,X}}(X_{\mathrm{K}} - X_{\mathrm{K'}})}{\Gamma_{\mathrm{r,T}}(T_{\mathrm{K}} + T_{\mathrm{K'}}) + \Gamma_{\mathrm{r,X}}(X_{\mathrm{K}} + X_{\mathrm{K'}})} = \frac{\rho_{\mathrm{T}} I_{\mathrm{T}} + \rho_{\mathrm{X}} I_{\mathrm{X}}}{I_{\mathrm{T}} + I_{\mathrm{X}}}. \tag{17}$$

Here the valley polarization of the trions ρ_{T} and excitons ρ_{X} are constant and their intensities I_{T} and I_{X} vary with the area of the weakly-doped region compared to the optical spotsize ($0 \leq A \leq 1$), according to

$$\begin{aligned} I_{\mathrm{X}} &= A \cdot I_{0,\mathrm{X}}, \\ I_{\mathrm{T}} &= (1 - A) \cdot I_{0,\mathrm{T}}. \end{aligned} \tag{18}$$

Here $I_{0,\mathrm{T}}$ and $I_{0,\mathrm{X}}$ are constants that indicate the trion and exciton photoluminescence when their associated regions would fill an entire optical spot. The total photoluminescence is given by

$$I = I_{\mathrm{T}} + I_{\mathrm{X}} = I_{0,\mathrm{T}} + A(I_{0,\mathrm{X}} - I_{0,\mathrm{T}}). \tag{19}$$

By substituting this expression into equation 17, we find that the ρ is inversely related to I for varying A

$$\rho = \frac{\rho_{\mathrm{X}} I_{0,\mathrm{X}} - \rho_{\mathrm{T}} I_{0,\mathrm{T}}}{I_{0,\mathrm{X}} - I_{0,\mathrm{T}}} + \frac{I_{0,\mathrm{X}} I_{0,\mathrm{T}} (\rho_{\mathrm{T}} - \rho_{\mathrm{X}})}{I_{0,\mathrm{X}} - I_{0,\mathrm{T}}} \cdot \frac{1}{I}. \tag{20}$$

This is a similar inverse relation as the one in equation 15 for a varying doping level. Note that this relation is exclusively inverse, and cannot explain any local maximum. However,

it is likely that the inverse decay of valley polarization in Figure 4c of the main text is a combination of 1) spatial averaging due to a diffraction limited optical spot and 2) local variations in doping.

Section S6. Schematic overview of the experimental setup

In Figure S6 we plot a schematic overview of the microscope used for the measurement of photoluminescence maps and emission spectra. We refer to the Methods section of the main text for the product details of each element.

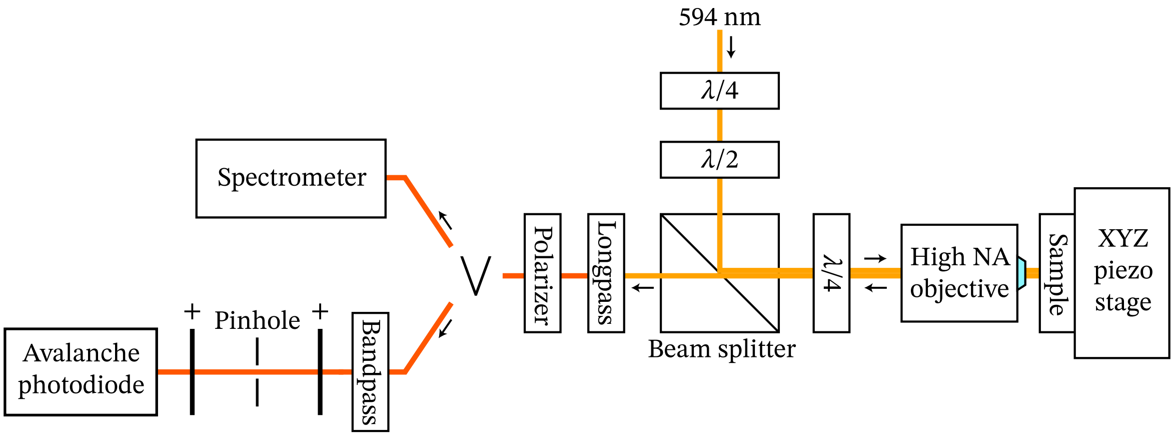

Figure S6: Overview of the measurement setup used in our experiments. The first quarter-lambda plate ($\lambda/4$) corrects for imperfections in the laser polarization and makes it perfectly linear, such that the excitation polarization can be controlled by turning the half-lambda plate ($\lambda/2$). A 10:90 beam splitter (R:T) separates the excitation from the detection. The polarization of the detection is controlled by the orientation of the second quarter-lambda plate relative to the transmission axis of the polarizer. The objective focuses the laser and collects the photoluminescence of the sample, which is positioned using an XYZ piezo stage. Two longpass filters eliminate the laser reflection. Depending on the orientation of a mirror on a computer-controlled flipmount (indicated by the V) the photoluminescence is detected by an avalanche photodiode (APD) or spectrometer. Before the emission is detected by the APD, it is filtered with a pinhole and bandpass filter.

References

(1) Zhuang, H. L.; Hennig, R. G. Computational Search for Single-Layer Transition-Metal Dichalcogenide Photocatalysts. *The Journal of Physical Chemistry C* **2013**, *117*, 20440–20445.

(2) Camacho-mendoza, R. L.; Zárate-hernández, L. A.; Vásquez-pérez, J. M.; Cruz-borbolla, J.; Alvarado-rodríguez, J. G.; Thangarasu, P. On the interaction of anisole and thioanisole derivatives with gold clusters studied by DFT. *Computational and Theoretical Chemistry* **2018**, *1126*, 54–64.

(3) Castellanos-Gomez, A.; Roldán, R.; Cappelluti, E.; Buscema, M.; Guinea, F.; van der Zant, H. S. J.; Steele, G. A. Local Strain Engineering in Atomically Thin MoS_2. *Nano Letters* **2013**, *13*, 5361–5366.

(4) Harats, M. G.; Kirchhof, J. N.; Qiao, M.; Greben, K.; Bolotin, K. I. Dynamics and efficient conversion of excitons to trions in non-uniformly strained monolayer WS_2. *Nature Photonics* **2020**, *14*, 324–329.

(5) Mak, K. F.; He, K.; Lee, C.; Lee, G. H.; Hone, J.; Heinz, T. F.; Shan, J. Tightly bound trions in monolayer MoS_2. *Nature Materials* **2013**, *12*, 207–211.

(6) Mitioglu, A. A.; Plochocka, P.; Jadczak, J. N.; Escoffier, W.; Rikken, G. L. J. A.; Kulyuk, L.; Maude, D. K. Optical manipulation of the exciton charge state in single-layer tungsten disulfide. *Phys. Rev. B* **2013**, *88*, 245403.

(7) Lien, D.-H.; Uddin, S. Z.; Yeh, M.; Amani, M.; Kim, H.; Ager, J. W.; Yablonovitch, E.; Javey, A. Electrical suppression of all nonradiative recombination pathways in monolayer semiconductors. *Science* **2019**, *364*, 468–471.

(8) Ross, J. S.; Wu, S.; Yu, H.; Ghimire, N. J.; Jones, A. M.; Aivazian, G.; Yan, J.;

Mandrus, D. G.; Xiao, D.; Yao, W.; Xu, X. Electrical control of neutral and charged excitons in a monolayer semiconductor. *Nature Communications* **2013**, *4*, 1474.

(9) Siviniant, J.; Scalbert, D.; Kavokin, A. V.; Coquillat, D.; Lascaray, J.-P. Chemical equilibrium between excitons, electrons, and negatively charged excitons in semiconductor quantum wells. *Phys. Rev. B* **1999**, *59*, 1602–1604.

(10) Zhang, W.; Tanaka, K.; Hasegawa, Y.; Shinokita, K.; Matsuda, K.; Miyauchi, Y. Bright and highly valley polarized trions in chemically doped monolayer MoS_2. *Applied Physics Express* **2020**, *13*, 35002.

(11) Robert, C.; Lagarde, D.; Cadiz, F.; Wang, G.; Lassagne, B.; Amand, T.; Balocchi, A.; Renucci, P.; Tongay, S.; Urbaszek, B.; Marie, X. Exciton radiative lifetime in transition metal dichalcogenide monolayers. *Phys. Rev. B* **2016**, *93*, 205423.

(12) Zhu, C. R.; Zhang, K.; Glazov, M.; Urbaszek, B.; Amand, T.; Ji, Z. W.; Liu, B. L.; Marie, X. Exciton valley dynamics probed by Kerr rotation in WSe_2 monolayers. *Phys. Rev. B* **2014**, *90*, 161302.
